# Comprehensive investigation of oncogenic driver mutations in Chinese non-small cell lung cancer patients

**DOI:** 10.18632/oncotarget.5549

**Published:** 2015-10-12

**Authors:** Rui Wang, Yang Zhang, Yunjian Pan, Yuan Li, Haichuan Hu, Deng Cai, Hang Li, Ting Ye, Xiaoyang Luo, Yiliang Zhang, Bin Li, Lei Shen, Yihua Sun, Haiquan Chen

**Affiliations:** ^1^ Department of Thoracic Surgery, Fudan University Shanghai Cancer Center, Shanghai, China; ^2^ Department of Oncology, Shanghai Medical College, Fudan University, Shanghai, China; ^3^ Department of Pathology, Fudan University Shanghai Cancer Center, Shanghai, China; ^4^ Shanghai Chest Hospital, Shanghai Jiao Tong University, Shanghai, China; ^5^ Institutes of Biomedical Sciences, Fudan University, Shanghai, China

**Keywords:** non-small cell lung cancer, driver mutations, ERBB, FGFR

## Abstract

**Purpose:**

To determine the frequency of driver mutations in Chinese non-small cell lung cancer (NSCLC) patients.

**Methods:**

Comprehensive mutational analysis was performed in 1356 lung adenocarcinoma, 503 squamous cell carcinoma, 57 adenosquamous lung carcinoma, 19 large cell carcinoma and 8 sarcomatoid carcinoma. The effect of EGFR tyrosine kinase inhibitors (TKIs) on *EGFR*-mutated lung adenocarcinoma patients after disease recurrence was investigated.

**Results:**

Mutations in *EGFR* kinase domain, *HER2* kinase domain, *KRAS*, *BRAF*, *ALK*, *ROS1* and *RET* were mutually exclusive. In lung adenocarcinoma cases “pan-negative” for the seven above-mentioned driver mutations, we also detected two oncogenic *EGFR* extracellular domain mutations (A289D and R324L), two *HER2* extracellular and transmembrane domain mutations (S310Y and V659E), one *ARAF* S214C mutation and two *CD74-NRG1* fusions. Six (1.2%) *FGFR3* activating mutations were identified in lung squamous cell carcinoma (five S249C and one R248C). There were three (15.8%) *EGFR* mutations and four (21.1%) *KRAS* mutations in large cell carcinoma. Three (37.5%) *KRAS* mutations were detected in sarcomatoid carcinoma. In *EGFR*-mutated lung adenocarcinoma patients who experienced disease recurrence, treatment with EGFR TKIs was an independent predictor of better overall survival (HR = 0.299, 95% CI: 0.172–0.519, *P* < 0.001).

**Conclusion:**

We determined the frequency of driver mutations in a large series of Chinese NSCLC patients. EGFR TKIs might improve the survival outcomes of *EGFR*-mutated lung adenocarcinoma patients who experienced disease recurrence.

## INTRODUCTION

Treatment strategies for non-small cell lung cancer (NSCLC) have been revolutionized since the identification of *EGFR* activating mutations which predict response to EGFR tyrosine kinase inhibitors (TKIs) in 2004 [1, 2]. Over the last decade, various oncogenic driver mutations have been identified in NSCLC, which enables this disease to be classified into clinically relevant molecular subgroups. Large phase III randomized clinical trials have proved the efficacy of targeted therapies over conventional cytotoxic chemotherapy for NSCLC patients harboring *EGFR* mutations [3–6] or *ALK* fusions [7]. In this study, we presented our sequencing results of a comprehensive panel of oncogenic driver mutations in a large prospective series of NSCLC patients who received surgical resection.

## RESULTS

### Frequency of oncogenic driver mutations in NSCLC histologic subtypes

**Figure 1:**
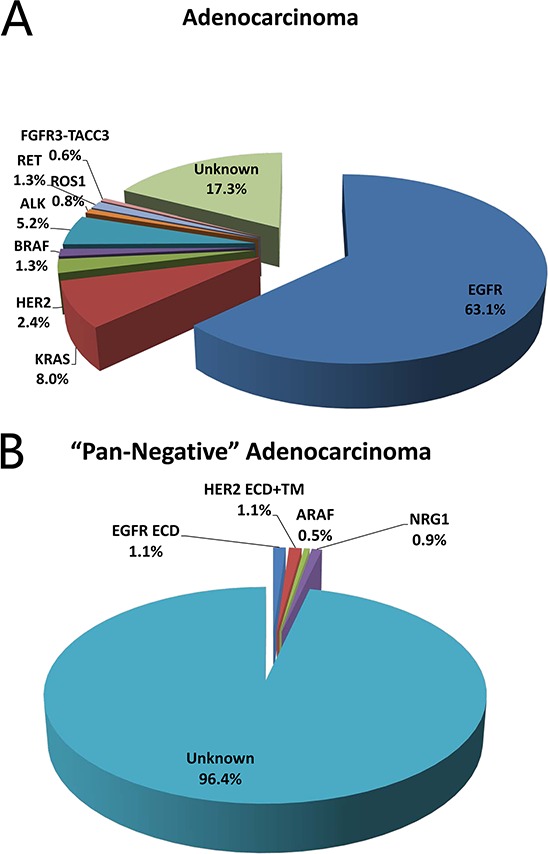
Frequency of driver mutations in lung adenocarcinoma **A.** and lung adenocarcinoma “pan-negative” for mutations in *EGFR* kinase domain, *KRAS*, *HER2* kinase domain, *BRAF*, *ALK*, *ROS1* and *RET* **B.**

A total of 1356 lung adenocarcinoma cases from April 2007 to May 2013 were sequenced for *EGFR* kinase domain mutations, *KRAS* mutations, *HER2* kinase domain mutations, *BRAF* mutations, *ALK* fusions, *ROS1* fusions, *RET* fusions and *AKT1* mutations. There were 855 (63.1%) *EGFR* kinase domain mutations (including 361 exon 19 deletions, 402 L858R and 92 other mutations), 108 (8.0%) *KRAS* mutations, 32 (2.4%) *HER2* kinase domain mutations (all were exon 20 insertion mutations), 18 (1.3%) *BRAF* mutations (5 V600E and 13 non-V600E mutations), 70 (5.2%) *ALK* fusions, 11 (0.8%) *ROS1* fusions and 17 (1.3%) *RET* fusions (Figure 1A). All the seven above-mentioned oncogenic driver mutations were mutually exclusive. We also identified 2 (0.1%) *AKT1* mutations, both were E17K mutations. One patient with *AKT1* E17K mutation also harbored *BRAF* V600E mutation; the other did not harbor any of the seven above-mentioned mutations. The identification of *FGFR* fusions has been reported in our previous study [8]. Six *FGFR3-TACC3* fusions were detected out of 1016 lung adenocarcinomas, accounting for a mutation rate of 0.6%.

In cases “pan-negative” for mutations in *EGFR*, *HER2*, *KRAS*, *BRAF*, *ALK*, *ROS1* and *RET*, we also sequenced for activating mutations in *EGFR* extracellular domain (ECD), *HER2* ECD and transmembrane domain, *ERBB3*, *ARAF* and *NRG1* (a total of 183 cases for ERBB family genes, and 219 cases for *ARAF* and *NRG1*) (Figure 1B). Oncogenic *EGFR* ECD mutations were detected in two cases (1.1%): one was A289D, and the other was R324L. One S310Y mutation and one V659E mutation was detected in *HER2* extracellular and transmembrane domain (1.1%), respectively. There was one (0.5%) *ARAF* S214C mutation. Two (0.9%) *CD74-NRG1* fusions were detected. No *ERBB3* activating mutations were detected.

**Figure 2:**
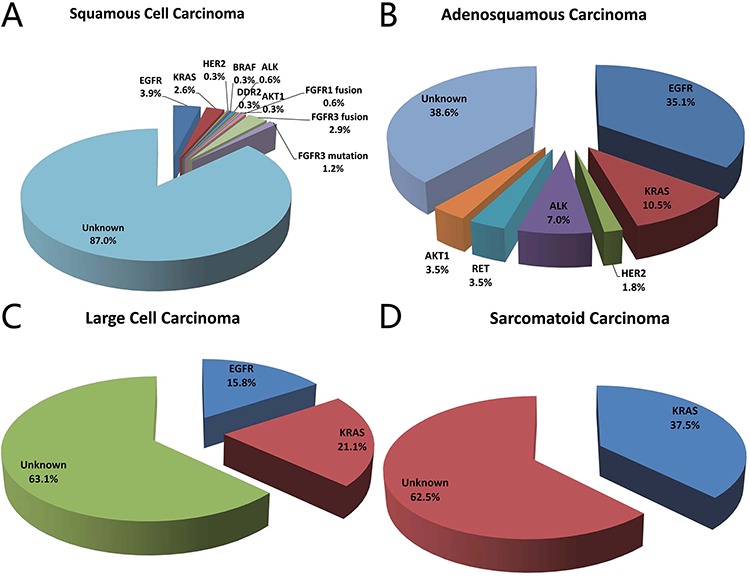
Frequency of driver mutations in lung squamous cell carcinoma **A.** adenosquamous carcinoma **B.** large cell carcinoma **C.** and sarcomatoid carcinoma **D.**

In lung squamous cell carcinoma, the mutation rate of *EGFR* (12 out of 310, 3.9%), *KRAS* (8 out of 310, 2.6%), *HER2* (1 out of 310, 0.3%), *BRAF* (1 out of 310, 0.3%), *ALK* (2 out of 310, 0.6%), *DDR2* (1 out of 310, 0.3%), *AKT1* (1 out of 310, 0.3%), *FGFR1* fusions (2 out of 312, 0.6%) and *FGFR3* fusions (9 out of 312, 2.9%) has been reported in our previous studies [8, 9]. We sequenced 503 lung squamous cell carcinoma resected from October 2007 to March 2013 for the prevalence of activating *FGFR2* and *FGFR3* mutations. Six (1.2%) *FGFR3* activating mutations were identified, including 5 S249C mutations and 1 R248C mutation (Figure 2A). No *FGFR2* activating mutations were detected.

Fifty-seven adenosquamous lung carcinoma resected between October 2007 to January 2013 were analyzed for mutations in *EGFR* kinase domain, *HER2* kinase domain, *KRAS*, *BRAF*, *ALK*, *RET* and *AKT1*. There were 20 (35.1%) *EGFR* mutations, 6 (10.5%) *KRAS* mutations, 1 (1.8%) *HER2* mutation, 4 (7.0%) *ALK* fusions, 2 (3.5%) *RET* fusions and 2 (3.5%) *AKT1* E17K mutations (Figure 2B).

We also sequenced 19 large cell carcinoma samples resected from November 2007 to May 2012 to detect mutations in *EGFR* kinase domain, *HER2* kinase domain, *KRAS*, *BRAF*, *ALK*, *RET* and *AKT1*. There were 3 (15.8%) *EGFR* mutations and 4 (21.1%) *KRAS* mutations (Figure 2C).

Eight sarcomatoid carcinoma were analyzed for the presence of *EGFR* kinase domain mutations, *KRAS* mutations, *HER2* kinase domain mutations, *BRAF* mutations, *ALK* fusions, *RET* fusions and *AKT1* mutations. Three (37.5%) *KRAS* mutations were detected, including 2 G12C and 1 G12V (Figure 2D).

### Clinicopathologic characteristics of NSCLC patients harboring *FGFR3* mutations, *AKT1* mutations, *EGFR* ECD mutations, *HER2* ECD and transmembrane domain mutations, *ARAF* mutations or *NRG1* fusions

All the 6 lung squamous cell carcinoma patients with oncogenic *FGFR3* mutations were male, and 5 of them were ever smokers. They all have tumors larger than 3 cm in diameter (mean: 5.4, range: 3.2–8.0). However, N2 disease was not found in any case (4 N0 and 2 N1). Both of the two adenocarcinoma cases with *CD74-NRG1* fusions were female never-smoking stage I invasive mucinous adenocarcinoma. Detailed clinicopathologic characteristics of patients with these rare mutations were listed in Table 1. Individual characteristics of patients harboring other mutations were showed in Supplementary Table S1.

**Table 1:** Individual patient data of non-small cell lung cancer harboring oncogenic *AKT1* mutations, *FGFR3* mutations, *EGFR* extracellular domain mutations, *HER2* extracellular and transmembrane domain mutations, *ARAF* mutations and *NRG1* fusions

No.	Gene	Mut	Histology	Sex	Age	Smoke	T (cm)	N	Stage
**1**	*AKT1*	E17K	AD	F	73	Never	1.5	N0	1a
**2**	*AKT1*	E17K	AD	M	30	Never	6	N1	2b
**3**	*AKT1*	E17K	AdSqLC	M	60	Ever	1.8	N0	1a
**4**	*AKT1*	E17K	AdSqLC	F	62	Never	5	N0	1b
**5**	*AKT1*	E17K	SCC	M	50	Ever	5.5	N1	2b
**6**	*FGFR3*	S249C	SCC	M	79	Ever	3.2	N0	1b
**7**	*FGFR3*	S249C	SCC	M	68	Ever	4.5	N1	2a
**8**	*FGFR3*	S249C	SCC	M	64	Ever	6.5	N0	2a
**9**	*FGFR3*	S249C	SCC	M	62	Ever	4.5	N0	1b
**10**	*FGFR3*	S249C	SCC	M	49	Never	8.0	N0	2b
**11**	*FGFR3*	R248C	SCC	M	59	Ever	5.5	N1	2b
**12**	*EGFR*	A289D	AD	F	64	Never	3	N0	1a
**13**	*EGFR*	R324L	AD	M	74	Ever	4.5	N2	3a
**14**	*HER2*	S310Y	AD	F	37	Never	3	N2	3a
**15**	*HER2*	V659E	AD	F	62	Never	0.7	N0	1a
**16**	*ARAF*	S214C	AD	F	51	Never	2.5	N0	1a
**17**	*NRG1*	CD74-NRG1	AD	F	69	Never	0.6	N0	1a
**18**	*NRG1*	CD74-NRG1	AD	F	62	Never	2.7	N0	1a

Abbreviations: Mut, mutations; T, tumor size; N, nodal status; AD, adenocarcinoma; AdSqLC, adenosquamous lung carcinoma; SCC, squamous cell carcinoma; F, female; M, male.

**Figure 3:**
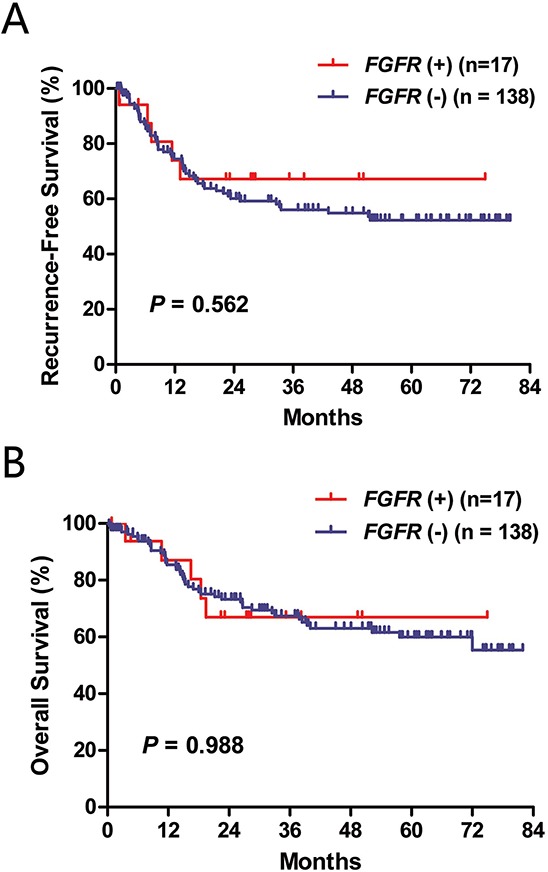
Recurrence-free survival **A.** and overall survival **B.** according to *FGFR* fusion and mutation status. *FGFR* (+), either *FGFR* fusion or *FGFR* mutation was positive; *FGFR* (−), both *FGFR* fusion and *FGFR* mutation were negative.

We then compared clinicopathologic characteristics between the 17 lung squamous cell carcinoma patients harboring *FGFR* fusions or *FGFR* mutations and 138 lung squamous cell carcinoma cases (both *FGFR* fusions and *FGFR* mutations were negative) from October 2007 to April 2010. Age, gender, smoking history, tumor size, nodal status and pathologic stage were not significantly different between the two groups (Table 2). RFS (*P* = 0.562) and OS (*P* = 0.988) were also comparable (Figure 3).

**Table 2:** Clinicopathologic features of lung squamous cell carcinoma harboring *FGFR* fusions or *FGFR* mutations

Variables	*FGFR* (+) (*n* = 17)	*FGFR* (−) (*n* = 138)	*P*
**Age (years)**	58.9	60.1	0.613
**Sex**			0.603
**Female**	0	10	
**Male**	17	128	
**Smoking history**			0.475
**Never**	1	23	
**Ever**	16	115	
**Tumor Size (cm)**			0.269
**Mean**	5.1	4.5	
**SD**	1.8	2.0	
**Nodal status**			1.000
**N0**	10	80	
**N1/N2**	7	58	
**Pathologic stage**			0.520
**I**	6	60	
**II/III**	11	78	

Abbreviations: *FGFR* (+), tumors harboring *FGFR* fusions or *FGFR* mutations; *FGFR* (−), both *FGFR* fusions and *FGFR* mutations were negative; SD, standard deviation.

### The effect of EGFR TKIs on *EGFR*-mutated lung adenocarcinoma patients after disease recurrence

**Figure 4:**
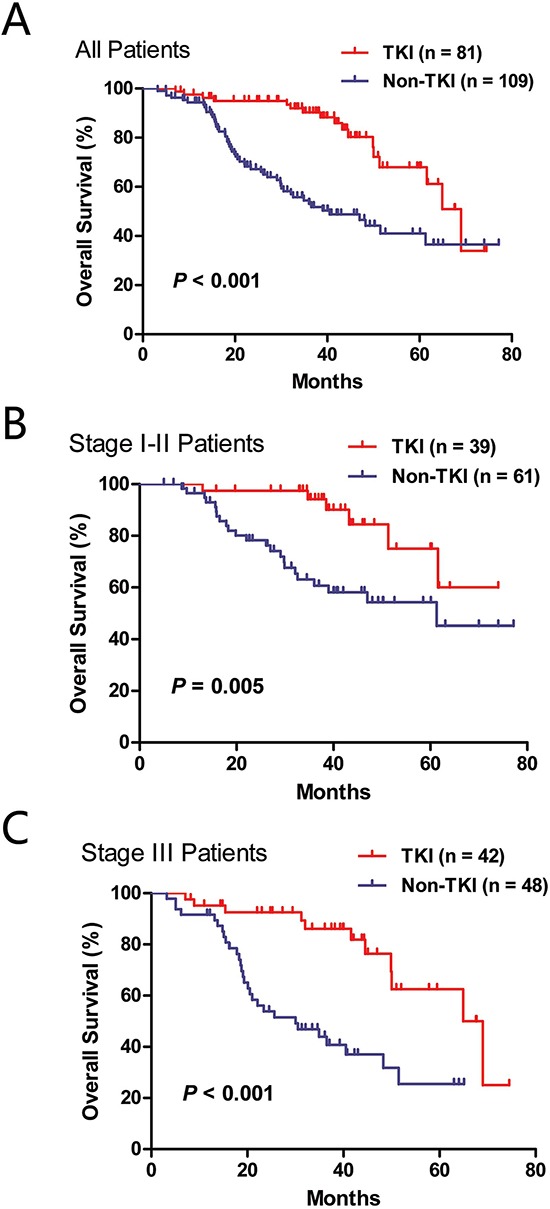
Overall survival of *EGFR*-mutated lung adenocarcinoma patients with or without the treatment of EGFR tyrosine kinase inhibitors (TKI) after disease recurrence **A.** all patients; **B.** stage I-II patients; **C.** stage III patients.

To ensure sufficient follow up, we included patients undergoing surgical resection from October 2007 to March 2012 for survival analysis. RFS of lung adenocarcinoma patients harboring *EGFR* mutations, *KRAS* mutations or *ALK* fusions were compared to that of wild-type patients (Supplementary Figure S1). No significant survival difference was found. A total of 190 lung adenocarcinoma patients with classic *EGFR* exon 19 deletions or L858R experienced disease recurrence. Eighty-one of these patients received EGFR TKIs (gefitinib or erlotinib) treatment after disease recurrence. Compared to those who did not receive TKIs, patients who were treated with TKIs had significantly better OS (*P* < 0.001) (Figure 4A). We further performed the survival analysis according to the initial pathologic stage (Figure 4B and 4C). Patients treated with targeted therapies had significantly better survival outcomes both in the stage I-II group (*P* = 0.005) and the stage III group (*P* < 0.001). Multivariate analysis adjusting for age, sex, smoking history and pathologic stage revealed that treatment with EGFR TKIs was an independent predictor of better OS (HR = 0.299, 95% CI: 0.172–0.519, *P* < 0.001).

## DISCUSSION

Using multiplexed assays of oncogenic drivers in lung cancers to select targeted drugs has been demonstrated to be feasible by the Lung Cancer Mutation Consortium (LCMC) [10]. Here, we performed a comprehensive analysis of oncogenic driver mutations in a large series of Chinese NSCLC patients.

*EGFR* (15.8%) and *KRAS* (21.1%) mutations were present in a considerable proportion in lung large cell carcinoma. De Pas and colleagues [11] reported a case of lung large cell carcinoma patient harboring *EGFR* mutation having dramatic response to gefitinib treatment. We further found that lung sarcomatoid carcinoma had a high frequency (37.5%) of *KRAS* mutations. Our results have implications for targeted therapy for patients with these rare histologic subtypes of NSCLC.

Liao and colleagues reported in 2013 the identification of inhibitor-sensitive oncogenic *FGFR2* and *FGFR3* mutations in lung squamous cell carcinoma from the Cancer Genome Atlas (TCGA) dataset [12]. They reported a mutation rate of 3% for each of the two genes. However, we screened more than 500 Chinese lung squamous cell carcinoma samples, and found that oncogenic *FGFR3* mutations were present in only 1.2% (5 S249C and 1 R248C). No oncogenic *FGFR2* mutations were detected in this series of patients. In addition to the 3.5% (11 out of 312) of *FGFR* fusions in lung squamous cell carcinoma [8], approximately 5% of Chinese lung squamous cell carcinoma could be defined by oncogenic alterations in the *FGFR* family genes. Through clinicopathologic analysis, we also found that patients with oncogenic *FGFR3* mutations were characterized by male, smokers, and larger tumor size without mediastinal lymph node metastasis.

Currently, the detection of mutations in *ERBB* family genes is mainly limited to the kinase domains of *EGFR* and *HER2*. However, the identification of oncogenic driver mutations in other sites of the *ERBB* family genes has continuously been reported [13–17]. For example, oncogenic *HER2* extracellular domain mutations were found in 0.8% (2 out of 258) of lung adenocarcinoma in the TCGA dataset [13]. Jaiswal and colleagues [14] reported oncogenic *ERBB3* mutations were present in approximately 1% of lung adenocarcinoma from a Western cohort. Here we found two *EGFR* ECD mutations (A289D and R324L), one *HER2* ECD mutation (S310Y), one *HER2* transmembrane domain mutation (V659E) and no *ERBB3* activating mutations, suggesting that oncogenic driver mutations in non-*EGFR* or *HER2* kinase domain of *ERBB* family genes do exist, but in a very small proportion of Chinese lung adenocarcinoma patients.

Imielinski and colleagues [18] reported that oncogenic and sorafenib-sensitive *ARAF* mutations were present in 1% of lung adenocarcinoma cases in the TCGA samples. *NRG1* fusions were also identified as novel oncogenic driver mutations in lung adenocarcinoma [19, 20]. The frequency of *NRG1* fusions was reported to be approximately 1.7% in lung adenocarcinomas from an Asian population [20]. Here, we found one *ARAF* mutation (S214C) and two *CD74-NRG1* fusions in 219 “pan-negative” lung adenocarcinoma cases. Both of the two cases with *NRG1* fusions were invasive mucinous adenocarcinoma, which was consistent with previous reports that *NRG1* fusions were characterized by invasive mucinous adenocarcinoma histology [19, 20].

Targeting oncogenic driver mutations has transformed the management of lung adenocarcinoma patients. Kris and colleagues [10] investigated the presence of oncogenic mutations of 10 genes in more than 1000 lung adenocarcinoma patients, and revealed that patients with an oncogenic driver and received genotype-directed therapy had significantly prolonged survival than those with an oncogenic mutation but did not receive genotype-directed therapy. Here, we investigated the effect of EGFR TKIs on *EGFR*-mutated lung adenocarcinoma patients after disease recurrence. Compared to those who did not receive TKIs, patients who were treated with TKIs had significantly better OS. Multivariate analysis showed the administration with EGFR TKIs was an independent predictor of better OS. The superiority of EGFR TKIs (including gefitinib, erlotinib and afatinib) over conventional chemotherapy in advanced lung adenocarcinoma has been well demonstrated in Phase III randomized trials [3–6, 21]. However, to the best of our knowledge, there is no clinical trial specifically investigating the efficacy of EGFR TKIs in lung cancer patients who have recurrent disease after surgical resection. Our results further supported the role of genotype-directed therapies for lung adenocarcinoma patients.

In conclusion, we determined the frequency of driver mutations in a large series of Chinese NSCLC patients. EGFR TKIs might improve the survival outcomes of *EGFR*-mutated lung adenocarcinoma patients who experienced disease recurrence.

## MATERIALS AND METHODS

### Patients and samples

From April 2007 to May 2013, lung tumors resected at the Department of Thoracic Surgery, Fudan University Shanghai Cancer Center were consecutively collected. Eligibility criteria included sufficient tissue for comprehensive mutational analysis and no neoadjuvant chemotherapy. After frozen tumor specimens were dissected into TRIzol (Invitrogen), total RNA were extracted per standard protocol (RNeasy Mini Kit; Qiagen, Hilden, Germany), and were subsequently reverse transcribed into cDNA using RevertAid First Strand cDNA Synthesis Kit (Fermentas, St Leon-Rot, Germany). Clinicopathologic data prospectively collected included age at diagnosis, gender, smoking history, pathologic TNM stage and tumor histology.

### Mutational analysis

Briefly, we designed primers to amplify *EGFR* (extracellular domain and kinase domain), *HER2* (extracellular domain, transmembrane domain and kinase domain), *ERBB3* (extracellular domain and kinase domain), *KRAS* (exons 2–3), *BRAF* (exons 11–15), *AKT1*, *ARAF*, *FGFR2* (extracellular domain and kinase domain) and *FGFR3* (extracellular domain) by PCR using cDNA. Direct dideoxynucleotide sequencing was used to analyze the amplified products. For newly identified mutations, we sequenced the germline DNA from the paired normal lung tissues to demonstrate they are somatically acquired. For the detection of *ALK*, *ROS1*, *RET*, *NRG1*, *FGFR1*, *FGFR2* and *FGFR3* fusions, we designed multiple pairs of primers to cover all the known fusion variants.

### Statistical analysis

Pearson's chi-squared test or Fisher's exact test was used to investigate correlations between two categorical variables. Independent sample *t*-test was used to assess associations between one categorical variable and one continuous variable. Kaplan-Meier method with log-rank test was used to compare recurrence-free survival (RFS) and overall survival (OS) in univariate analysis. Cox proportional hazards regression (forward likelihood ratio model) was used to estimate the hazard ratio (HR) and 95% confidence interval (CI) in multivariate survival analysis. The statistical analysis was conducted in SPSS 16.0 (SPSS Inc, Chicago, Ill). All tests were two tailed, and statistical significance was set at *P* < 0.05.

### Ethics statement

This study was conducted in line with the Helsinki Declaration, and was approved by the Institutional Review Board of the Fudan University Shanghai Cancer Center. Written informed consent was obtained from each patient to allow their biological samples to be genetically analyzed. The experimental protocol of this study was performed strictly in accordance to the guidelines.

## SUPPLEMENTARY FIGURE AND TABLE




