# Supplementary material for: Comprehensive investigation of oncogenic driver mutations in Chinese non-small cell lung cancer patients
Source: Oncotarget. 2015 Oct 12;6(33):34300–8. doi: 10.18632/oncotarget.5549 (PMC4741453; doi:10.18632/oncotarget.5549)
Supplement: Supplementary file 1 [file oncotarget-06-34300-s001.pdf]

SUPPLEMENTARY FIGURE AND TABLE

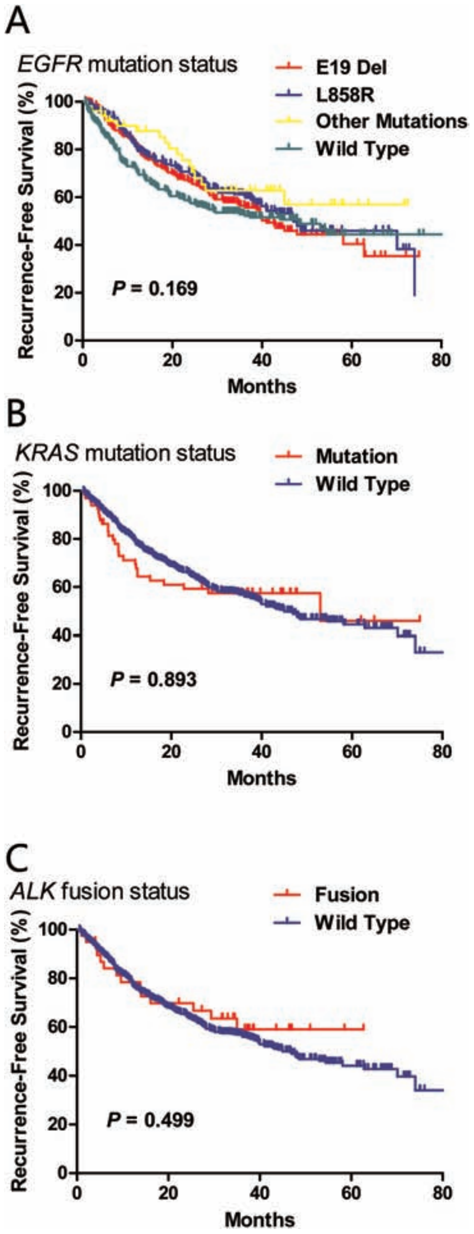

Supplementary Figure S1: Recurrence-free survival of lung adenocarcinoma patients harboring *EGFR* mutations **A.** *KRAS* mutations **B.** or *ALK* fusions **C.** Abbreviations: E19 Del, *EGFR* exon 19 deletions.

Supplementary Table S1: Individual patient data of non-small cell lung cancer harboring *EGFR* tyrosine kinase domain mutations, *KRAS* mutations, *HER2* tyrosine kinase domain mutations, *BRAF* mutations, *DDR2* mutation, *ALK* fusions, *ROS1* fusions, *RET* fusions and *FGFR* fusions.
